# Absolute Configuration of Beer's Bitter Compounds**

**DOI:** 10.1002/anie.201208450

**Published:** 2012-12-13

**Authors:** Jan Urban, Clinton J Dahlberg, Brian J Carroll, Werner Kaminsky

**Affiliations:** a Department of Chemistry, University of Washington, Box 351700, Seattle, WA 98195 (USA); b KinDex Therapeutics, 800 Fifth Avenue, Suite 4100, Seattle, WA 98104 (USA)

**Keywords:** beer, configuration determination, hops, humulone, isomerization

The science and art of making beer, likely the oldest liquid fermented by humans, stretches over millennia. Production typically involves boiling beer wort together with hops, which acts as a natural preservative,^1^ but the generated iso-α-acids are known to be prone to decomposition,^2^, ^3^ and consequently, more stable reduced hops extracts, such as the tetrahydro-iso-α-acids, have been developed. These latter compounds are separately produced and frequently added to beer to achieve a consistent level of bitter taste. Scheme 1 gives an overview of the iso-α-acids formed by heat-induced isomerization.

**Scheme 1:**
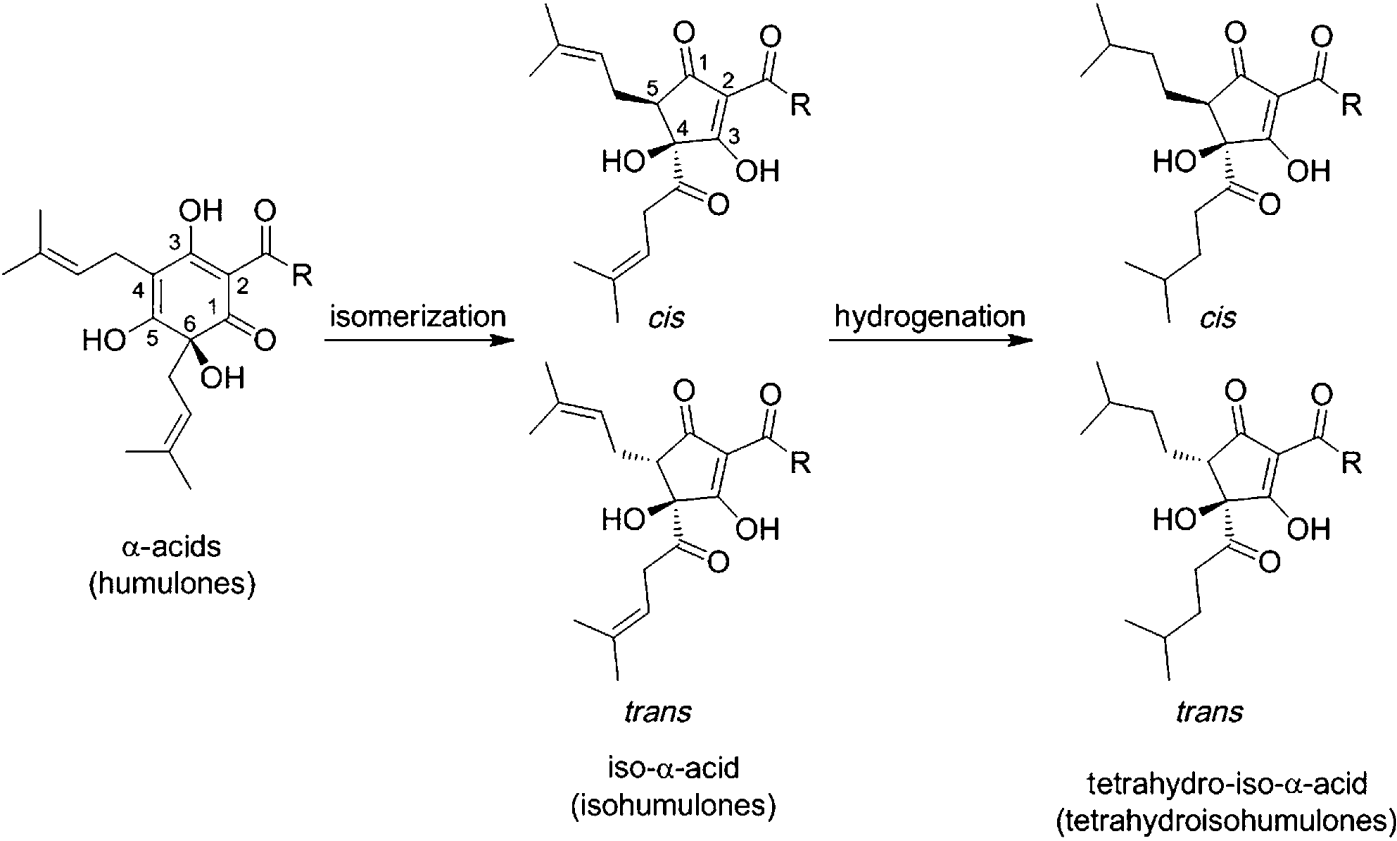
Hops bitter acids. Humulones: R=isobutyl, cohumulones: R=isopropyl, adhumulones: R=*sec*-butyl.

Herein, we determined the absolute configuration of several *cis* and *trans* iso-α-acids by X-ray crystallography. We show how we unequivocally assigned the chiral center in (−)-humulone to be (6*S*) and give absolute structures for several of its derivatives, most of which contradict the general perception circulating through the literature since 1970.^4^

Typically, the predominant α-acid is humulone, a phloroglucinol derivative with two prenyl groups and one isovaleryl group as side chain. The process of isomerization involves contraction of the six-membered α-acid ring (through acyloin rearrangement) to form the five-membered iso-α-acid ring with two chiral centers,^5^ resulting in *cis* and *trans* diastereomers. Photo-induced isomerization is stereospecific and can be used to produce pure *trans* iso-α-acids.

The isomerization process has been recognized for over 80 years,^6^–^8^ yet the absolute configuration of carbon atoms 4 and 5 of the iso-α-acids (Scheme 1) have remained speculative. For the most predominant member of the iso-α-acid family, isohumulone, the *cis* and *trans* isomerization products, differing at carbon atom 4, were described preliminarily almost 50 years ago (Scheme 1).^9^ Ultimately, the absolute configuration of (4*R*, 5*S*) for (+)-*cis*-isohumulone was inferred according to Horeau’s method of partial decoupling. The absolute configuration of (−)-tetrahydrohumulone was inferred using the Cotton effect, which relates spectral details in an optical rotary dispersion curve to the configuration of a molecule, among other things.^4^, ^10^ However, contradictive and predominantly unsupported or inconclusive configurational assignments are reported as well.^11^–^13^

Claims that beer and the bittering acids found in beer are beneficial when consumed in moderation have accumulated over time, including positive effects on diabetes,^14^–^16^ forms of cancer,^17^–^20^ and inflammation,^21^ and even linking reduced iso-α-acid derivatives to weight loss.^22^–^24^ Some of these derivatives affect one illness,^21^, ^25^–^28^ whereas others, differing only in the configuration of carbon atoms 4 and 5 of the iso-α-acids (Scheme 1), are ineffective.^29^ In addition, it was discovered that different grades of bitterness can be related to opposite enantiomers of tetrahydro-iso-α-acids.^30^

However, the degree of bitterness of the isomerized bittering acids has yet to be related to a specific structure–function relationship after decades of confusion over the configuration of iso-α-acids, and in general, specifics of the isomerization process need to be resolved. Moreover, preservation of configuration during thermal isomerization has in the past been assigned to the ring carbon atom with the 3-methylbutyl side chain (see Scheme 1, C5 of the iso-α-acids, derived from C4 of the α-acids),^6^, ^7^, ^10^ which was opposed by other reports,^11^, ^31^, ^32^ thus demonstrating the state of confusion about the underlying hops chemistry.

In order to unequivocally derive the absolute configuration of the iso-α-acids, we sought to use X-ray diffraction on a salt of (+)-*cis*-isohumulone or a suitable derivative and record the optical rotation of the compounds to ensure accurate literature comparison.

Please note that the isomerization process results in a multitude of very similar compounds, which need to be separated from each other, purified, and unambiguously characterized, requiring an elaborate procedure (for details see the Supporting Information).

As crystal salts we chose those containing either a heavy atom (to enable anomalous X-ray scattering for adequate phase resolution to define the configuration of a structure) or a compound of known absolute configuration to serve as an internal reference.

Empirically, the vast number of useful salt combinations was greatly reduced by the inability to grow X-ray quality crystals. The initial success in elucidating the absolute configuration occurred with a purified ‘tetrahydro’ derivative (compound **1**, (+)-*cis*-tetrahydroisohumulone), synthesized from (+)-*cis*-isohumulone through heterogeneous catalytic hydrogenation.^29^

The absolute configuration, which was determined by X-ray analysis on a potassium salt (+)**-1 a**), indicates that the configuration of compound **1** (4*S*, 5*R*) is opposite to the originally reported configuration of the unsaturated precursor (+)-*cis*-isohumulone.^10^ Considering that the inversion of both asymmetric centers (C4 and C5 in Figure 1) is unlikely to occur during mild catalytic hydrogenation, the initial assignment found in the literature would appear to be incorrect. In an attempt to verify this observation, additional crystallization experiments were undertaken with compounds related to **1**, resulting in the (−)-cinchonidine crystal salts of (+)-*cis*-tetrahydroisocohumulone (**2**), (+)-*cis*-tetrahydroisoadhumulone (**3**), and (+)-*cis*-isohumulone (**4**; Table 1, salts **2 b**, **3 b**, **4 b**).

**Fig 1:**
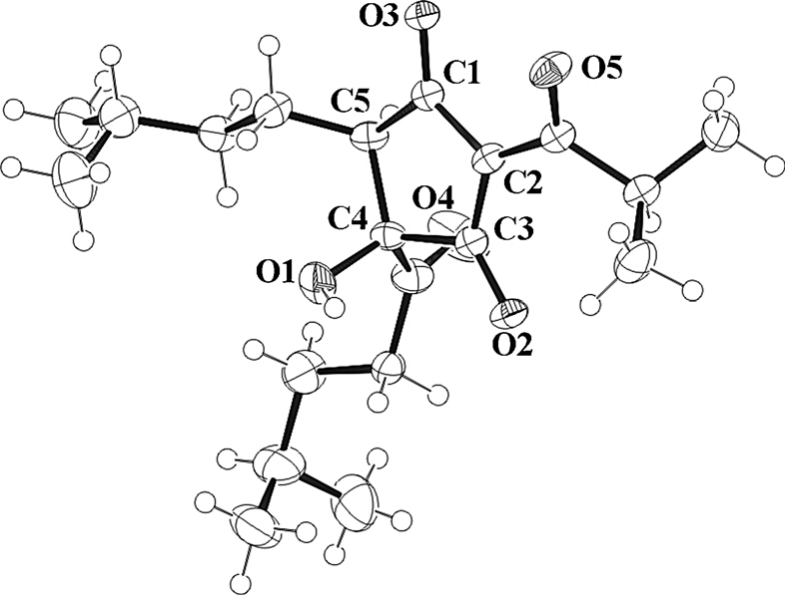
Structural details of (+)-*cis*-tetrahydroisocohumulone (**2**). The conserved stereocenter during isomerization is C4, while *cis* and *trans* iso-α-acids differ stereochemically at C5, which is nonchiral in the precursor α-humulone molecule. Related structures are discussed in Table 1 and in the Supporting Information.

**Table 1:** Crystallized compounds suitable for X-ray structure determinations and details on measurement of specific rotation.

Compound	Free acid parent molecule, specific rotation	Salt compound, counterion, specific rotation
**1** (4*S*, 5*R*)	(+)-*cis*-tetrahydroisohumulone, +25.1 (+26.7)^[a]^	(+)-**1 a**, potassium, +71.5
(−)-**1** (4*R*, 5*S*)	(−)-*cis*-tetrahydroisohumulone^[b]^	(−)-**1 a**, potassium, −60.5
**2** (4*S*, 5*R*)	(+)-*cis*-tetrahydroisocohumulone, +35.3 (+35.6)^[a]^	**2 b**, (−)-cinchonidine
**3** (4*S*, 5*R*)	(+)-*cis*-tetrahydroisoadhumulone^[b]^	**3 b**, (−)-cinchonidine
**4** (4*S*, 5*R*)	(+)-*cis*-isohumulone^[b]^	**4 b**, (−)-cinchonidine
**5** (6*S*)	(−)-humulone, −197.7 (−212)^[a]^	**5 c**, (1*R*, 2*R*)-1,2-diaminocyclohexane
**6** (4*S*, 5*S*)	(−)-*trans*-tetrahydroisohumulone, −11.2 (−12.4)^[a]^	**6 d**, (+)-cinchonine

[a] Ting, Goldstein.^31^ [b] Configuration of compound determined by HPLC analysis on a chiral stationary phase (see the Supporting Information). Measurement of specific rotation: *c*=1.0, MeOH (see the Supporting Information), reference values in parentheses.

As expected, the absolute configurations of these compounds were consistent with the one we determined for compound **1** (4*S*, 5*R*). For comparison, we added the unnatural enantiomer of **1** to this series, (−)-*cis*-tetrahydroisohumulone ((−)-**1**), characterized here as (4*R*, 5*S*).

Encouraged by these findings, we focused on the stereochemical assignment of the α-acid precursor (−)-humulone. Although neither a potassium nor (−)-cinchonidine salt were amenable to crystallization, we discovered that (−)-humulone (**5**) formed a small and weakly diffracting crystal (**5 c**) with *trans*-(1*R*, 2*R*)-(−)-diaminocyclohexane, thus enabling determination of the configuration of (−)-humulone as (6*S*). This finding is consistent with our initial assignment of (+)-*cis*-tetrahydroisohumulone, which again is opposite to the configuration found in most references.

Finally, we were able to crystallize (−)-*trans*-tetrahydroisohumulone (**6**) with (+)-cinchonine (salt **6 d**). The resulting structure has unequivocally a (4*S*, 5*S*) configuration, and anomalous X-ray diffraction (enabled through the presence of chloroform solvent molecules) confirmed the result. Table 1 summarizes all findings.

In light of these discoveries, the isomerization of humulone to isohumulones proceeds with a net retention of configuration from the tertiary alcohol in (6*S*)-(−)-humulone to the α-hydroxy ketone (C4) in (4*S*, 5*R*)-(+)-*cis*-isohumulone, while configuration at C5 (Figure 1) is not determined. These results are in contrast with the proposed isomerization mechanism found in most reports, which assume that the conserved stereocenter is on C5, while *cis* and *trans* differ stereochemically at C4. Considering that the oxygen atoms possess negative charges during the isomerization, one might imagine the chelation of two vicinal oxygen atoms to a divalent cation, a process that is known to accelerate the rate of isomerization, while limiting decomposition.

Excessive beer consumption cannot be recommended to propagate good health, but it has been demonstrated that isolated humulones and their derivatives can be prescribed with documented health benefits.^21^ The absence of correct stereochemical assignment for these compounds has prevented verification of the actual species responsible for biological activity. Now that the stereochemistry for these compounds has been confirmed and methods have been developed to substantiate the configuration of new entities in this series (see the Supporting Information), future work on their biological activities should be greatly accelerated.

The utility of X-ray diffraction as the ultimate and preferred method to obtain unequivocal answers regarding absolute stereochemical questions (such as those above) has once more been demonstrated. One question remains: to what extent can one trust those assignments derived indirectly through Horeau’s method of partial decoupling and the Cotton Effect in optical rotary dispersion, now that we have discovered a case where these methods have failed the scientific community?

## Notes

Detailed facts of importance to specialist readers are published as ”Supporting Information”. Such documents are peer-reviewed, but not copy-edited or typeset. They are made available as submitted by the authors.
