# Supplementary material for: Absolute Configuration of Beer's Bitter Compounds
Source: Angew Chem Int Ed Engl. 2012 Dec 13;52(5):1553–5. doi: 10.1002/anie.201208450 (PMC3563212; doi:10.1002/anie.201208450)
Supplement: Supplementary file 1 [file anie0052-1553-sd1.pdf]

Angewandte Chemie
Eine Zeitschrift der Gesellschaft Deutscher Chemiker

Supporting Information

© Wiley-VCH 2013

69451 Weinheim, Germany

Absolute Configuration of Beer's Bitter Compounds**

*Jan Urban, Clinton J. Dahlberg, Brian J. Carroll, and Werner Kaminsky**

anie_201208450_sm_miscellaneous_information.pdf

Supplementary Information

Table of Contents

SI.1 MATERIALS AND METHODS 2
SI1.1 General considerations 2
SI1.2 Analytical Instrumentation 2
SI1.3 Countercurrent Chromatography Instrumentation 5
SI.2 Material Preparation and characterization 6
SI2.1 Generalized Procedures 6
SI2.2 Specific Preparations 7
SI3. X-RAY STRUCTURES 12
References 21

SI.1 MATERIALS AND METHODS

SI.1.1 General considerations:

All solvents and buffers used for HPLC analyses were purchased through VWR International and were HPLC or ACS grade. Chemical Water was obtained from a Barnstead Nanopure Infinity Ultrapure system maintained within our laboratories. Commercially available hops extracts (Hopsteiner) were used for the isolation/preparation of individual hop acids. The chemicals used as bases for crystallization were purchased from Sigma Aldrich Chemical and used without further purification. Authentic hops standards were provided by the American Society of Brewing Chemists. Further structural confirmation was provided via specific rotation, melting point, NMR, UV, and UPLC-MS/MS when compared to preceding literature reports as available.

SI1.2 Analytical Instrumentation

NMR spectra were recorded at 298 K on a Bruker AV300, AV500, DRX500 and/or Varian INOVA 500, or 600 spectrometers. ^{1}H-NMR chemical shifts are reported versus TMS and referenced to residual solvent. Elemental analyses and specific rotation measurements were performed by Robertson Microlit Laboratories (Madison, NY) utilizing a Perking-Elmer 341-Polarimeter. All reactions/purifications were monitored by HPLC and/or UPLC-MS/MS. HPLC analyses were performed using a Shimadzu Prominence HPLC system (Shimadzu Scientific Instruments, Inc.). Suitable non-chiral reverse-phase chromatography and chiral normal-phase chromatography methods used for chemical and/or reaction evaluation by HPLC are presented in Tables SI-1 and SI-2 respectively.

Table SI-1. Description of HPLC methods of analysis for iso-α acids (IAA), α acids (AA), and tetrahydro iso-α acids (THIAA) using a Phenomenex Gemini-NX C18 (3 μm) column (40 ^{o}C).

	**IAA**		**AA**		**THIAA**	
**Column (mm)**	4.6x250		2x150		2x150	
**A Solvent**	50 mM NH_4Ac, pH 9.5		50 mM NH_4Ac, pH 9.5		50 mM NH_4Ac, pH 9.5	
**B Solvent**	6:4 MeCN:MeOH		MeOH		MeOH	
**Flow Rate (mL/min)**	1.5		0.4		0.4	
	**Time**	**%B***	**Time**	**%B***	**Time**	**%B***
**Program (minutes)**	0	40	0	60	0	55
	17	40	10	60	20	55
	18	95	13	70	21	95
	20	95	14	95	23	95
	22	40	16	95	25	55
	24	40	18	60	30	55
			22	60		

*% of B Solvent in the mobile phase

Table SI-2. Description of chiral HPLC methods of analysis for iso-α acids (IAA), α acids (AA), and tetrahydro iso-α acids (THIAA) using a Regis (S,S)-Whelk-01 (5 μm) column (38 °C).

	IAA	AA	THIAA
**Column (mm)**	4.6x250	4.6x250	4.6x250
**A Solvent**	0.1% TFA in IPA	100 mM NH_4Ac in IPA	100 mM NH_4Ac in IPA
**B Solvent**	Hexanes	Hexanes	Hexanes
**Flow Rate (mL/min)**	1.5	1.5	1.5
**Isocratic Conditions***	98	80	87

*% of B Solvent in the mobile phase

UPLC-MS/MS analyses were performed using a Shimadzu Nexera UPLC system (Shimadzu Scientific Instruments, Inc.) connected to an API-2000 MS/MS (ABSciex). All UPLC conditions can be seen in Table SI-3. All fragmentation information was acquired using information dependent acquisition (IDA) in negative mode (M-H) and the fragmentation methods can be provided upon request. A representative chromatogram for the UPLC-MS/MS analysis of the ASBC tetrahydro iso-α acid standard (ICS-T2) can be seen in Figure SI-1, with a table of fragmentations and relative retention times (Table SI-4) for the corresponding structures (Figure SI-2).

Table SI-3. Description of UPLC methods of analysis iso-α acids (IAA), α acids (AA), and tetrahydro iso-α acids (THIAA) using a Waters Acquity UPLC BEH C18 (1.7 μm) column (40 °C).

	IAA		AA		THIAA	
**Column (mm)**	3x150		3x150		3x150	
**A Solvent**	15 mM NH_4Ac, 1mM $(NH4)_2CO_3$, pH 9.5		15 mM NH_4Ac, 1mM $(NH4)_2CO_3$, pH 9.5		15 mM NH_4Ac, 1mM $(NH4)_2CO_3$, pH 9.5	
**B Solvent**	MeOH		MeOH		MeOH	
**Flow Rate (mL/min)**	0.5		0.5		0.4	
	**Time**	**%B***	**Time**	**%B***	**Time**	**%B***
**Program (minutes)**	0	48	0	55	0.0	60
	30	55	30	65	10.0	60
	30.5	95	30.5	95	26.0	68
	31.0	95	31.0	95	26.1	95
	31.1	48	31.1	55	26.6	95
	35.0	48	35.0	55	26.7	60
					30.0	60

*% of B Solvent in the mobile phase

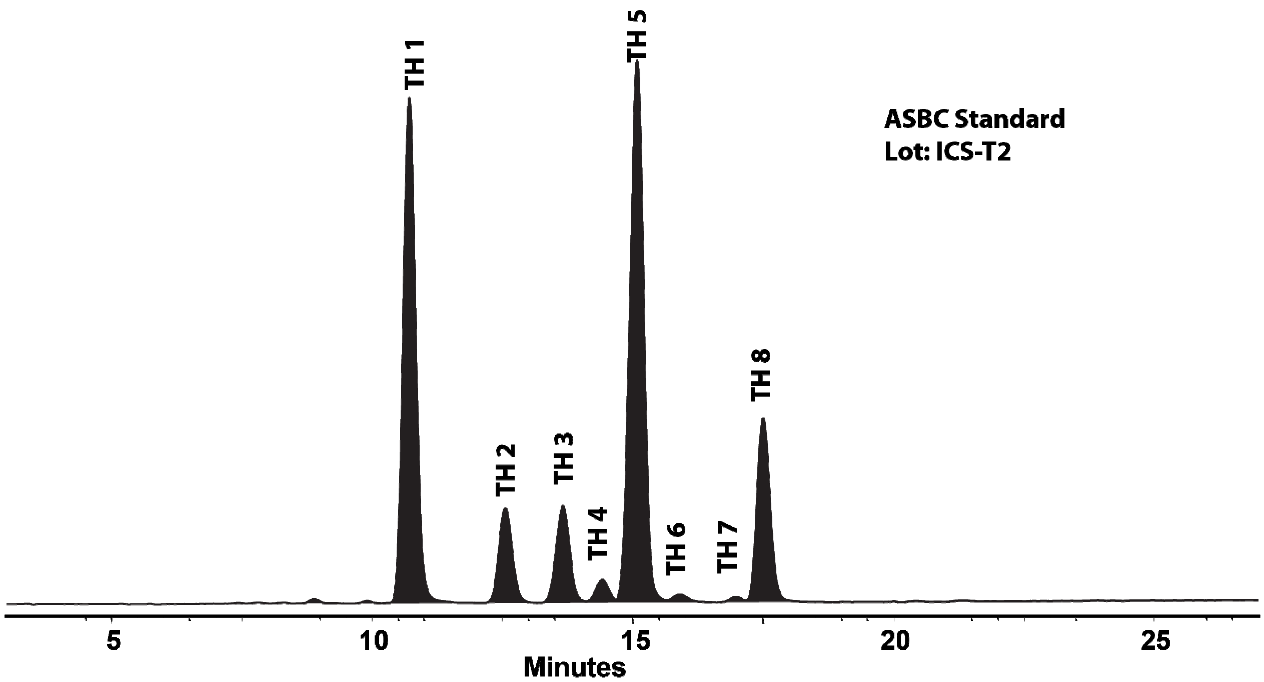

Figure SI-1: UV_{254} trace of the American Society of Brewing Chemists calibration standard (ICS-T2)

Table SI-4: Component Identification of the American Society of Brewing Chemists calibration standard (ICS-T2)

		PDA Description		*MS Description*								
**Component**	**Relative Retention Time (min)**	**Peak Area % (254nm)**	**Lambda Max (nm)**	***Parent Ion*** **[M-H]**	***MS/MS Fragmentation*** **Neutral Loss [M-H]**							
TH 1	-4.3	32.7	255	351.4	18	44	98	112	116		142	140
TH 2	-2.5	6.5	255	351.3	18	44	98	112	116		142	140
TH 3	-1.4	6.9	255	365.3	18	44	98	112	116	126	142	154
TH 4	-0.6	1.6	254	365.3	18	44	98	112	116	126	142	154
TH 5	0.0	38.7	254	365.5	18	44	98	112	116	126	142	154
TH 6	0.8	0.6	255	365.2	18	44	98	112	116	126	142	154
TH 7	1.9	0.3	254	365.2	18	44	98	112	116	126	142	154
TH 8	2.4	12.4	254	365.3	18	44	98	112	116	126	142	154

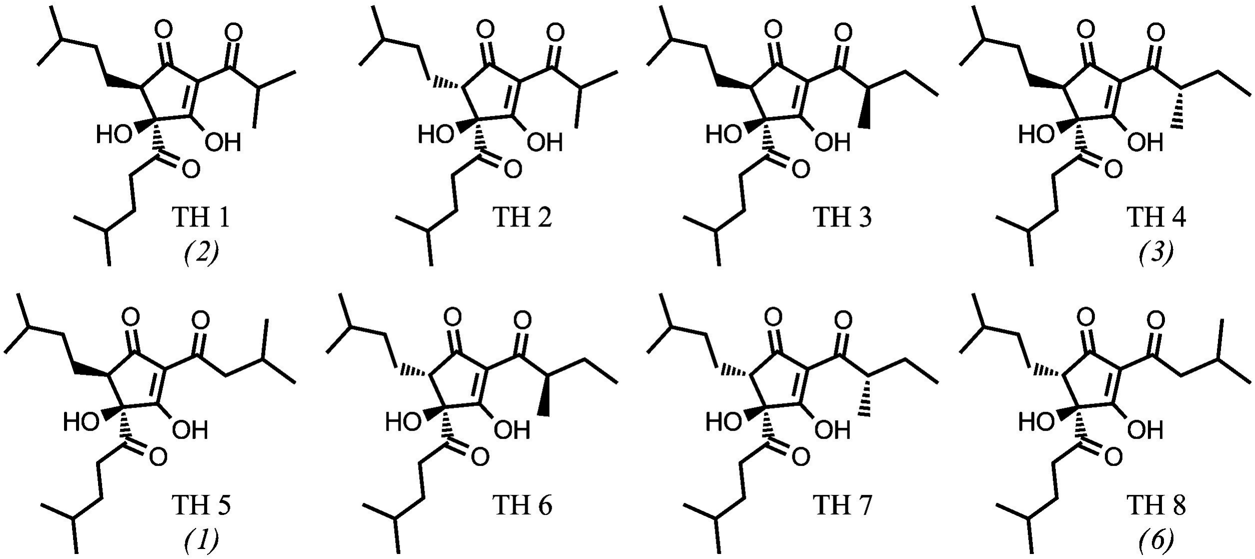

Figure SI-2: Components of the American Society of Brewing Chemists calibration standard (ICS-T2)

SI 1.3 Countercurrent Chromatography Instrumentation

All countercurrent chromatography (CCC) experimentation was conducted using a J-type synchronous three-coiled planetary motion CCC-1000 (PharmaTech Research Corp.) as the column; this instrument possesses 810 mL of total column volume. This CCC column was coupled with Shimadzu preparative liquid chromatography instrumentation. Standard protocols for CCC were used over the course of the experimentation ,[S4-S6] and specific details of the methods used can be found in Table SI-5.[S7,S 9] Further information is available upon request.

Table SI-5: Description of countercurrent chromatography methods of purification for the iso-α acids (IAA), α acids (AA), and tetrahydro iso-α acids (THIAA).

**CCC Method**	**Hop Extract**	**pH**	**Solvent System**				**Buffer**	
			**Volume Ratio**					
			n-C_6H_{14}	EtOAc	CH_3OH	Buffer	**Type**	**Molarity $[M]_{Aq}$**
**1**	**AA**	8.7	9	1	9	1	$(NH_4)Ac$	0.25
**2**	**IAA**	5.0	1			1	$(NH_4)Ac$	0.25
**3**		4.1	7	3	5	5	$(NH_4)HCO_2$	0.50
**4**	**THIAA**	7.0	1			1	$(NH_4)H_2PO_4$	0.25
**5**		5.6	7	3	5	5	$(NH_4)Ac$	0.50

SI.2 Material Preparation and characterization

SI2.1 Generalized Procedures

SI2.1.1 Removal of *trans* iso-α acids *via* β-cyclodextrin complexation

Hops isomerized resin extract (45g, 0.12 mol) was mixed with 200mL of water and following the addition of 13mL (0.12 mol) of 9.2M KOH over 5 minutes, this formed a homogeneous solution. In a separate flask, 141g (0.12 mol) of β-cyclodextrin were mixed with 1.1L of water and heated to 70 °C with mixing until the solution became homogenous/ translucent. The two solutions were slowly combined using a dropping funnel over 30 minutes. Following full addition, the mixture was removed from heat and allowed to come to room temperature at which point it was transferred to 4 °C storage for a period of 3 days. A precipitate formed during chill stabilization, and the supernatant was separated using centrifugation (1000 g, 10 minutes, 4 °C). The pellet was washed with 500 mL of water and the centrifugation was repeated; both aqueous supernatants were combined in a 4L separatory funnel. Ethanol (800 mL) was added to the supernatant, followed by 100mL of 1N HCl to bring the pH to ~1; without the ethanol a difficult emulsion forms. The supernatant was extracted three times with 200mL of hexanes and the extract was subsequently washed with water and brine to remove any residual β-cyclodextrin. Water (50mL) and 9.2M KOH (0.08mol, 10mL) were added in order to adjust to pH >8, followed by extraction of the organic layer three times with water (50 mL each time). The aqueous extract was collected in a 500mL Erlenmeyer flask and with vigorous stirring 12.4g (0.10 mol) of magnesium sulfate were slowly added to form a shelf-stable magnesium salt precipitate with the *cis* iso-α acids. The precipitate was collected via vacuum filtration, further dried via lyophilization and 21g were stored for future use. The yield and diastereoselectivity can be adjusted based on the equivalents of cyclodextrin used.[S8]

SI2.1.2 Catalytic Hydrogenation

Isohumulone (~700 mg, 2 mmol) was mixed with water and one equivalent of KOH (1.25 M aq. solution); a clear solution formed. To this solution was added an aqueous solution of magnesium sulfate (1.2 eq). A precipitate formed immediately, which was filtered, washed with water and dried on high vacuum overnight. This material was dissolved in methanol, 10% Pd on carbon was added (0.1 eq) and the reaction mixture was stirred under hydrogen (1 atm) until reaction completion (typical reaction time of 2 hours). After reaction completion, the catalyst was filtered off and the solvent was evaporated; this magnesium salt is well suited for storage. To liberate the free acid, material was suspended in 1M aq. HCl and the free acid was extracted to dichloromethane, the resulting dichloromethane extract was dried with sodium sulfate and evaporated.

Alternatively, isohumulone was dissolved in methanol and 1 equivalent of magnesium oxide was added. The resulting mixture was stirred for 30 minutes at which time most of the magnesium oxide dissolved. To this mixture was added Pd catalyst and the same steps as in the previous procedure were followed. The yield for either hydrogenation reaction typically ranges between 80-95%.

SI2.1.3 **Differential pH Extraction**

Hops CO_2 extract (101g,~0.16 mol of alpha acids) was dissolved in 100mL of dichloromethane in a 250mL Erlenmeyer flask. When the solution was homogeneous, it was transferred to a 1L separatory funnel and the flask was washed with an additional 100mL of dichloromethane. Water (200mL) was added to the separatory funnel along with 10mL (0.09mol) of 9.2 M KOH to bring the aqueous solution to pH 8. The organic layer was extracted twice with 200mL of water. An additional 200mL of water and 4mL (0.04mol) of 9.2M KOH were added and the pH was maintained at approximately 8.5. The final 200mL aqueous extraction used 5mL (0.05mol) of 9.2M KOH, for a total of 0.18 moles of KOH or approximately 1.1eq relative to the alpha acids presumed present in the extract. The aqueous extracts were combined and back-extracted one time to remove any excess beta acids. The dicholoromethane solutions were combined and concentrated *in vacuo* to produce a mixture of beta acids and essential hop oils. The aqueous extract was acidified to pH 2 using sulfuric acid and extracted three times with 100mL of dichloromethane, concentrated *in vacuo* to isolate 70g of alpha acids in ≥98% homogeneity in an apparent quantitative yield.

SI2.2 Specific Preparations

SI2.2.1 Preparation of 1: (+)-*cis* tetrahydro isohumulone: ((4S,5R)-3,4-dihydroxy-2-(3-methylbutanoyl)-5-(3-methylbutyl)-4-(4-methylpentanoyl)cyclopent-2-en-1-one).

Cis tetrahydro isohumulone was purified using countercurrent chromatography (CCC) according to a reported procedure from a commercially available hops extract.[S7] Analytical data confirmed ≥95% homogeneity following CCC purification.

Free acid: Anal. Found: C, 68.83, H, 9.43. $C_{21}H_{34}O_5$ requires C, 68.82, H, 9.35.

m.p. 46 °C (45-47 °C (*1*)), optical rotation +25.1, c=1.0, MeOH (+26.7)[S1]

^{1}H NMR (600 MHz, METHANOL-d_4) δ ppm 0.86 - 0.90 (m, 12 H) 0.92 - 0.96 (m, 6 H) 1.24 (dd, *J*=10.55, 6.59 Hz, 1 H) 1.22 - 1.28 (m, 1 H) 1.39 - 1.43 (m, 2 H) 1.48 - 1.53 (m, 2 H) 1.61 - 1.72 (m, 2 H) 2.08 - 2.12 (m, 1 H) 2.65 - 2.80 (m, 4 H) 3.08 (dd, *J*=7.47, 6.15 Hz, 1 H)

^{13}C NMR (126 MHz, METHANOL-d_4) δ ppm 21.36, 21.41, 21.42, 21.45, 21.49, 24.36, 25.68, 27.32, 27.95, 31.77, 35.30, 36.37, 45.80, 50.42, 87.28, 110.34, 196.81, 199.57, 204.78, 210.78;

The purified material was converted to the potassium salt by reaction with 1 equivalent of potassium hydroxide, and was recrystallized from water to increase purity and remove discoloration. After achieving sufficient homogeneity, the potassium salt was further recrystallized to render crystals of sufficient dimension for X-ray diffraction experiments.

Potassium salt (**+1a**): Anal. Found: C, 61.79, H, 8.18, K 9.28. C16H27O43 K8 requires C, 61.23, H, 8.27, K, 9.50.

m.p. 141 °C, optical rotation +71.5, c=1.0, MeOH

Water (450 µL) and (+)-*cis* tetrahydro isohumulone potassium salt (49.5 mg) were added to a 4 mL vial, and the vial was sealed with a cap and heated using a thermal heating block (70°C) until dissolution occurred. The solution was allowed to cool to 35 °C over a two hour period. During this time, *in situ* formation of crystals was observed. The mixture was allowed to stand at room temperature (22 °C) for eight hours, at which point the formation of additional crystals was observed. An individual crystal suitable for X-ray analysis was identified, carefully removed from solution, mounted, and submitted for X-ray diffraction analysis.

SI2.2.2 Preparation of (-1): (–)-*cis* tetrahydro isohumulone: ((4R,5S)-3,4-dihydroxy-2-(3-methylbutanoyl)-5-(3-methylbutyl)-4-(4-methylpentanoyl)cyclopent-2-en-1-one).

A mixture of alpha acids (65 g) was dissolved in 200ml of limonene, bubbled with Ar for 30 min and heated at 145 °C for 120 minutes, at which point the reaction mixture was cooled to 50 °C, solvent was evaporated and the degree of racemization was evaluated and found to be 3% ee (enantiomeric excess). The racemic alpha acids were isolated using lead-acetate precipitation procedure[S3] and further purified by CCC using the procedure outlined in Table S-5. 4 (±)-Humulone (4.34 g, 12.0 mmol) was dissolved in 200mL of isopropanol and stirred. (1R,2R)-4-Cyclohexene-1,2-diamine (1.41g , 12.6 mmol) was added slowly to the racemic mixture. Crystals began forming within 1 hour at room temperature, and the mixture was placed in the refrigerator overnight (4 °C chill-stabilization). The crystals were collected via filtration, and were found to be enriched in (–)-humulone (35% ee). The mother liquor was concentrated *in vacuo*, followed by the addition of 20mL of isopropanol for further recrystallization in order to recover additional (–)-humulone (35% ee) and enrich the mother liquor in (+)-humulone (85% ee). (+)-Humulone (1.43g , 4.0mmol) was combined with 1.0 mL of water and heated to 81 °C. While the mixture stirred, 273mg (2.3mmol) of magnesium sulfate were added followed by the slow addition of 430µL (4.0mmol) of 9.2M KOH to form a hard resin precipitate. The mixture was heated and stirred for a total of 7 hours at approximately 80 °C. Upon completion, the water was removed via pipette from the vial (leaving the resinous precipitate), and 10mL of methanol was used to dissolve the remaining material. Magnesium oxide (256mg) was added and the mixture was stirred and filtered on a 0.2µm syringe filter into 2 separate and equal volume stocks. Stock 1 had 34mg of additional magnesium oxide and 200mg of 10% Pd/C added, and it was stirred under hydrogen (1 atm) for 1 hour. Stock 2 had 47mg of magnesium oxide and 187mg of 10% Pd/C added, and it was stirred for 2.5 hours under hydrogen (1 atm). The pH was brought to 1-2 using sulfuric acid, and the resulting mixture was filtered to remove any precipitates. Following CCC purification,[S7] 351mg (1.0 mmol, 25% yield from (+)-humulone) of (–)-*cis* tetrahyrdo isohumulone) was isolated for crystallization. Analytical data confirmed the purified material to be ≥95% (–)-*cis* tetrahydro isohumulone.

The purified material was converted to a potassium salt by reacting with 1 equivalent of potassium hydroxide, and was recrystallized from water to increase purity. After achieving sufficient homogeneity, the potassium salt was further recrystallized to render crystals of sufficient dimension for X-ray diffraction experiments.

Potassium salt (**-1a**): Anal. Found (Calc for 8M+3 H2O, formula corresponding to crystal structure): C61.42, H, 8.67. $C_{168}H_{270}O_{43}K_8$ requires C, 61.32, H, 8.27.

m.p. 143 °C, optical rotation -60.5, c=1.0, MeOH

Water (110 µL) and the potassium salt of (–)-*cis* tetrahydro isohumulone (11.7 mg) were added to a 4 mL vial, the vial was sealed with a cap and heated using a thermal heating block (56°C) until dissolution occurred. The solution was allowed to cool to 43 °C for a few hours and a seed crystal was used to induce crystallization. The mixture was allowed to stand at room temperature (22 °C) until taken for X-ray analysis. An individual crystal suitable for X-ray analysis was identified, carefully removed from solution, mounted, and submitted to X-ray diffraction analysis.

SI2.2.3 Preparation of 2: (+)-*cis* tetrahydro isocohumulone: (4S,5R)-3,4-dihydroxy-5-(3-methylbutyl)-4-(4-methylpentanoyl)-2-(2-methylpropanoyl)cyclopent-2-en-1-one

The *trans* iso-α-acids were removed via β-cyclodextrin complexation (found in the generalized procedures section, [S8] from 45g of a hops isomerized resin extract. The *cis* material was further purified via CCC[9] in order to isolate *cis* isocohumulone. *Cis* isocohumulone was reduced via catalytic hydrogenation and repurified *via* CCC [S7] to ≥95% homogeneity.

Free acid: Anal. Found: C, 68.09, H, 9.11. $C_{20}H_{32}O_5$ requires C, 68.15, H, 9.15.

m.p. 49-50 °C (50-52 °C), optical rotation +35.3, c=1.0, MeOH (35.6) [S1]

Cinchonidine salt of (+)-*cis* tetrahydro isocohumulone: (4S,5R)-3,4-dihydroxy-5-(3-methylbutyl)-4-(4-methylpentanoyl)-2-(2-methylpropanoyl)cyclopent-2-en-1-one

1 Equivalent of each of (+)-*cis* tetrahydro isocohumulone (77.0 mg) and (–)-cinchonidine (51.9 mg, 1.00 eq.) were mixed in ^{i}Pr-OH (200 uL) and briefly heated to form a solution. ^{t}BuOMe (200 uL) was added and the resulting solution was left to crystallize at room temperature. Following three days at room temperature, the *in situ* formation of crystals was observed in this solution. An individual crystal suitable for X-ray analysis was identified, carefully removed, mounted and submitted for X-ray diffraction analysis.

Cinchonidine salt (**2b**): Anal. (Calc 2M + ^{t}BuOMe, formula corresponding to crystal structure) Found: C, 72.14, H, 8.42, N, 4.09. $C_{83}H_{120}N_4O_{13}$ requires C, 71.90, H8.75, N, 4.05.

m.p. 118 °C.

SI2.2.4 Preparation of 3: (+)-*cis* tetrahydro isoadhumulone: (4S,5R)-3,4-dihydroxy-2-[(2S)-2-methylbutanoyl]-5-(3-methylbutyl)-4-(4-methylpentanoyl)cyclopent-2-en-1-one

The *trans* iso-α-acids were removed via β-cyclodextrin complexation (found in the generalized procedures section, [S8] from 45g of a hops isomerized resin extract. The remaining *cis* material was further purified via CCC (9) in order to isolate *cis* isoadhumulone. *Cis* isoadhumulone was then reduced via catalytic hydrogenation and purified via CCC [S7] to ≥95% homogeneity.

Cinchonidine salt of (+)-*cis* tetrahydro isoadhumulone (4S,5R)-3,4-dihydroxy-2-[(2S)-2-methylbutanoyl]-5-(3-methylbutyl)-4-(4-methylpentanoyl)cyclopent-2-en-1-one

1 Equivalent each of (+)-*cis* tetrahydro isoadhumulone (50.0 mg) and (–)-cinchonidine (39.9 mg, 0.99 eq.) was added to ^{i}Pr-OH (200 uL) in a 4 ml amber colored vial and briefly heated (in a heating block set at 60-70 °C) to form a solution. Ether (200 uL) was added and the solution was left to form crystals at room temperature. The solution was sealed under a septum. The septum was pierced with a (Gauge16) needle to enable a gradual evaporation of solvent. Following three days at room temperature, the *in situ* formation of crystals was observed in this solution. An individual crystal suitable for X-ray analysis was identified, carefully removed, mounted and submitted for X-ray diffraction analysis.

Cinchonidine salt (**3b**): Anal. (Calc as 2M+0.6 iPrOH+0.4 Et2O, formula corresponding to crystal structure) Found: C, 70.68, H, 8.35, N, 3.98. $C_{83.4}H_{120.8}N_4O_{13}$ requires C70.57, H, 8.58, N, 3.95.

m.p. 149 °C.

SI2.2.5 Preparation of 4: (+)-*cis* isohumulone: (4S,5R)-3,4-dihydroxy-2-(3-methylbutanoyl)-5-(3-methylbut-2-en-1-yl)-4-(4-methylpent-3-enoyl)cyclopent-2-en-1-one

Hops isomerized resin extract (45g) had the trans- iso-α-acids removed via β-cyclodextrin complexation (found in the generalized procedures section.[8] The remaining *cis* material was

further purified via CCC[S7] in order to isolate *cis* isohumulone confirmed to be ≥95% homogeneous following purification.

Cinchonidine salt of (+)-*cis* isohumulone: (4S,5R)-3,4-dihydroxy-2-(3-methylbutanoyl)-5-(3-methylbut-2-en-1-yl)-4-(4-methylpent-3-enoyl)cyclopent-2-en-1-one

1 Equivalent each of (+)-*cis* isohumulone (99.4 mg) and (–)-cinchonidine (80.0 mg, 0.99 eq.) was mixed in ^{i}Pr-OH (100 uL) and briefly heated to form a solution. ^{t}BuOMe (500 uL) was added and the solution was left to form crystals at room temperature. Following three days at room temperature, the *in situ* formation of crystals was observed in this solution. An individual crystal suitable for X-ray analysis was identified, carefully removed, mounted and submitted for X-ray diffraction analysis.

Cinchonidine salt (**4b**): Anal. (Calc as M+0.3 H2O). Found: C, 72.65, H, 7.80, N, 4.28. $C_{40}H_{52.6}O_{6.3}$requires C 72.53, H, 8.02, N, 4.23.

m.p. 149 °C

SI2.2.6 Preparation of 5: (–)-humulone: (6*S*)-3,5,6-trihydroxy-2-(3-methylbutanoyl)-4,6-bis(3-methylbut-2-en-1-yl)cyclohexa-2,4-dien-1-one

(–)-Humulone (**5**) was isolated from commercially available hops CO_2 extract via a differential pH extraction, (found in the generalized procedures section), followed by CCC purification of the resultant alpha acid fraction [S10] to ≥95% homogeneity.

m.p. 66 °C (66 °C), [S1] optical rotation -197.7, c=1.0, MeOH (-212) [S1]

(1R,2R)-(-)-diaminocyclohexane salt of (–)-humulone: (6*S*)-3,5,6-trihydroxy-2-(3-methylbutanoyl)-4,6-bis(3-methylbut-2-en-1-yl)cyclohexa-2,4-dien-1-one

6.0 mg of (-)-N-humulone was mixed with (1R,2R)-(-)-diaminocyclohexane in MeOH. Solution was evaporated and redissolved in tBuOMe. It was left to evaporated slowly over several days, while crystals were formed.

1,2-Diamincyclohexane salt (**5c**): Anal. Found: C 68.05, H 9.56, N, 5.77. $C_{27}H_{44}N_2O_5$ requires C, 68.05, H, 9.30, N, 5.88.

m.p. 144 °C.

SI2.2.7 Preparation of 6: (–)-*trans* tetrahydro isohumulone: ((4S,5S)-3,4-dihydroxy-2-(3-methylbutanoyl)-5-(3-methylbutyl)-4-(4-methylpentanoyl)cyclopent-2-en-1-one)

The alpha acids were isolated from a commercially available hops CO_2 extract via a differential pH extraction (found in the generalized procedures section). A 450mm jacketed borosilicate glass immersion well was wrapped with approximately 17 meters of 2.7mm inner diameter FEP tubing and was used with a 450W medium pressure mercury immersion lamp to produce a flow photoreactor. [S11] The isolated alpha acids (2.1g) were dissolved in 100mL of methanol and run through the flow reactor at 5mL/min, for 2 hours, recycling the effluent until the reaction was complete. The resulting free acid mixture of *trans*- iso-α-acids was reduced via catalytic hydrogenation and purified *via* CCC to produce *trans* tetrahydro isohumulone[S7] in ≥95% homogeneity.

Free acid: Anal. Found: C, 68.05, H, 9.56, N, 5.77. $C_{27}H_{44}N_2O_5$ requires C, 68.04, H, 9.30, N, 5.88.

m.p. 76 °C (78-81 °C), [S1] optical rotation -11.2, c=1.0, MeOH (-12.4) [S1]

^{1}H NMR (500 MHz, METHANOL-d_4) δ ppm 0.88 - 0.95 (m, 12 H) 0.98 (dd, *J*=16.30, 6.68, Hz, 6 H) 1.18 - 1.26 (m, 1 H) 1.37 - 1.49 (m, 4 H) 1.50 - 1.60 (m, 2 H) 1.87 - 1.97 (m, 1 H) 2.09 - 2.19 (m, 1 H) 2.68 - 2.75 (m, 1 H) 2.75 - 2.86 (m, 4 H);

^{13}C NMR (126 MHz, METHANOL-d_4) δ ppm 21.26, 21.31, 21.36, 21.37, 21.49, 21.58, 22.19, 26.28, 27.25, 27.93, 31.35, 36.70, 37.04, 44.14, 47.10, 47.27, 47.44, 47.61, 47.79, 47.95, 48.12, 191.45, 196.44, 207.67, 210.23;

Cinchonine salt of (–)-*trans* tetrahydro isohumulone (**6d**): ((4S,5S)-3,4-dihydroxy-2-(3-methylbutanoyl)-5-(3-methylbutyl)-4-(4-methylpentanoyl)cyclopent-2-en-1-one)

(–)-*Trans* tetrahydro isohumulone (54.3 mg) was mixed with cinchonine (43.0 mg, 1 eq) in MeOH and the resulting solution was evaporated. The resulting solid salt was suspended in hexane and chloroform was added until complete dissolution occurred. The solution was left open to allow the solvent to slowly evaporate over several days, at which time crystals formed. An individual crystal suitable for X-ray analysis was identified, carefully removed, mounted and submitted to X-ray diffraction analysis. The remaining crystals were filtered, dried at high vacuum and submitted for elemental analysis.

Cinchonine salt: Anal. Anal. (Calc. as M+0.15$CHCl_3$) Found: C71.26, H, 8.35, N, 4.15. $C_{40.15}H_{56.1}Cl_{0.45}N_2O_6$ requires C, 71.04, 8.34, N, 4.13.

m.p. 137-139 °C.

SI3. X-RAY STRUCTURES

Methodology and instrumentations

Colorless crystals of about one-third of a mm in diameter were mounted on glass capillaries with oil. Data were collected on a Bruker APEX II single crystal X-ray diffractometer, Mo-radiation (collection temperature, see Table SI6). Crystal-to-detector distance was 40 mm and exposure time was 10 to 120 seconds per degree for all sets. The scan width was 0.5°. Data collection completeness to 25° in ϑ was above 98% in all cases. Further details are given in Table SI-6 below.

The data were integrated and scaled using SAINT, SADABS within the APEX2 software package by Bruker .[S12] Solution by direct methods (SHELXS, SIR97) [S13,S14] produced a complete heavy atom phasing model consistent with the proposed structure. The structures were completed by difference Fourier synthesis with SHELXL97[S15,S16] Scattering factors are from Waasmair and Kirfel. [S17] Hydrogen atoms were placed in geometrically idealised positions and constrained to ride on their parent atoms with C---H distances in the range 0.95-1.00 Angstrom. Isotropic thermal parameters (U_{eq}) were fixed such that they were $1.2U_{eq}$ of their parent atom's Ueq for CH's and $1.5U_{eq}$ of their parent atom's U_{eq} in the case of methyl groups. All non-hydrogen atoms were refined anisotropically by full-matrix least-squares. The weighting schemes were optimized by the values suggested by SHELXL after each refinement.

CCDC 913165 - 913171 contains the supplementary crystallographic data for this paper. These data can be obtained free of charge from the Cambridge Crystallographic Data Centre via www.ccdc.cam.ac.uk/data_request/cif.

SI3.1 X-ray structure of Compound +1a, potassium salt of (+)-cis n- tetrahydro iso α-acid

This structure, described in[S2] is added for completion. The asymmetric unit for this structure consists of 4 negatively charged molecules of (**1**) coordinating with 4 potassium cations. Two potassiums are bridged by a single water molecule, another disordered water coordinates with the remaining two cations. Ketones of the organic moieties coordinate with the cations leading to a close packing pattern. The absolute configuration was obtained from anomalous scattering (Absolute structure parameter = 0.04(4))

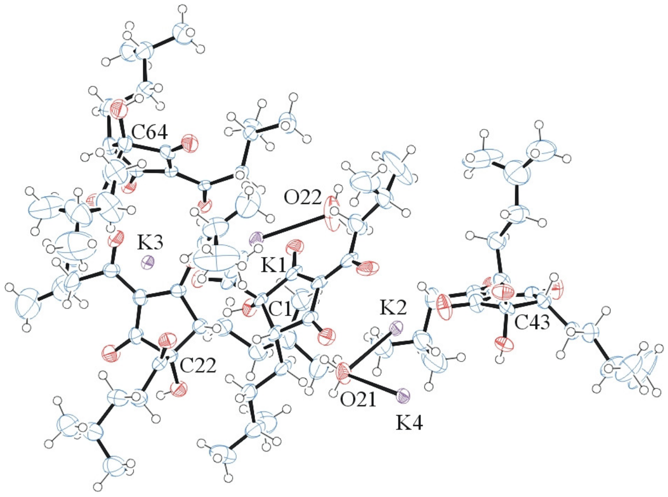

Figure SI-3. ORTEP representations of the asymmetric unit in the structure for compound **1** with displacement ellipsoids at the 50% probability level.

Table SI-6. Diffraction details of the structures provided. The absolute configuration for anions of compounds **2 – 5** were derived from the known chirality of the cations.

compound	**+1a**	**-1a**	**2b**	**3b**	**4b**	**5c**	**6d**
Empirical formula	C168 H270 K8 O43	C168 H270 K8O43	C83 H120 N4 O13	C83 H120 N4 O13	C40 H52 N2 O6	C27 H44 N2 O5	C41 H57 Cl3 N2 O6
Formula weight	3290.64	3290.64	1381.83	1381.83	656.84	476.64	780.24
Temperature (K)	110(2)	110(2)	110(2)	110(2)	110(2)	292(2)	100(2)
Wavelength (Å)	0.71073	0.71073	0.71073	0.71073	0.71073	0.71073	0.71073
Crystal system	Orthorhombic	Orthorhombic	Monoclinic	Monoclinic	Monoclinic	Orthorhombic	Monoclinic
Space group	$P\ 2_1\ 2_1\ 2$	$P\ 2_1\ 2_1\ 2$	$P\ 2_1$	$P\ 2_1$	$P\ 2_1$	$P\ 2_1\ 2_1\ 2_1$	$P\ 2_1$
Unit cell dim.							
a (Å)	23.3110(9)	23.2614(16)	17.3908(6)	17.4871(6)	14.474(2)	6.6424(12)	13.6570(12)
b (Å)	28.9052(12)	28.8798(22)	11.8605(4)	12.2205(4)	9.5817(13)	17.292(4)	10.4637(9)
c (Å)	13.6845(5)	13.6741(11)	20.4103(7)	20.0825(7)	14.579(2)	24.070(6)	14.6463(14)
β(°)	90	90	111.0900(10)	113.581(2)	118.624(7)	90	95.342(5)
Volume ($Å^3$)	9220.7(6)	9186.1(15)	3927.9(2)	3933.3(2)	1774.8(5)	2764.7(11)	2083.9(3)
Z	2	2	2	2	2	4	2
Density (cal. Mg/m^3)	1.185	1.185	1.168	1.167	1.229	1.145	1.243
Abs.coeff.(mm^{-1})	0.258	0.258	0.078	0.078	0.082	0.078	0.266
F(000)	3548	3548	1500	1500	708	1040	832
Crystal size (mm^3)	0.37 x 0.30 x 0.28	0.32 x 0.30 x 0.18	0.30 x 0.27 x 0.05	0.24 x 0.21 x 0.06	0.30 x 0.03 x 0.01	0.60 x 0.01 x 0.01	0.20 x 0.13 x 0.03
ϑmin / ϑmax (°)	2.05 / 25.35	2.05 / 25.34	1.92 / 28.32	1.99 / 26.44	1.59 / 25.55	2.06 / 25.54	1.50 / 25.51
Reflections collected	68211	208296	71085	114408	11710	14317	58994
Independent reflect.	16681	16769	10175	8453	3452	2931	7692
R(int)	0.0386	0.2922	0.0416	0.0545	0.1728	0.2542	0.0437
Completeness to ϑ=25°	98.4 %	99.4 %	99.4 %	99.3 %	99.3 %	99.9 %	99.8 %
Max. / min. transmiss.	0.9312 / 0.9106	0.9312 / 0.9106	0.9961 / 0.9770	0.9953 /0.9816	0.9992 / 0.9759	0.9992 / 0.9546	0.9921 / 0.9487
Data/ restr./param.	16681 / 0 / 1019	16769 / 0 / 1019	10175 / 92 / 1057	8453 / 34 / 917	3452 / 13 / 441	2931 / 3 / 316	7692 / 49 / 513
Goodness-of-fit on F^2	1.079	1.007	1.015	1.016	0.957	0.948	1.055
R indices [I>2σ(I)]							
R1, wR2	0.0694, 0.1704	0.0891, 0.1669	0.0448, 0.1007	0.0563, 0.1410	0.0641, 0.1094	0.0672, 0.0814	0.0418, 0.0902
(all data)							
R1, wR2	0.0795, 0.1823	0.2097, 0.2187	0.0626, 0.1098	0.0734, 0.1536	0.1649, 0.1399	0.2974, 0.1312	0.0581, 0.0982
Flack parameter	0.04(4)	0.11(6)	N/A	N/A	N/A	N/A	0.02(6)
diff. peak / hole(e.$Å^{-3}$)	0.949 / -0.424	0.770 / -0.391	0.433 / -0.248	0.678 / -0.487	0.303 / -0.338	0.188 / -0.202	0.239 / -0.256

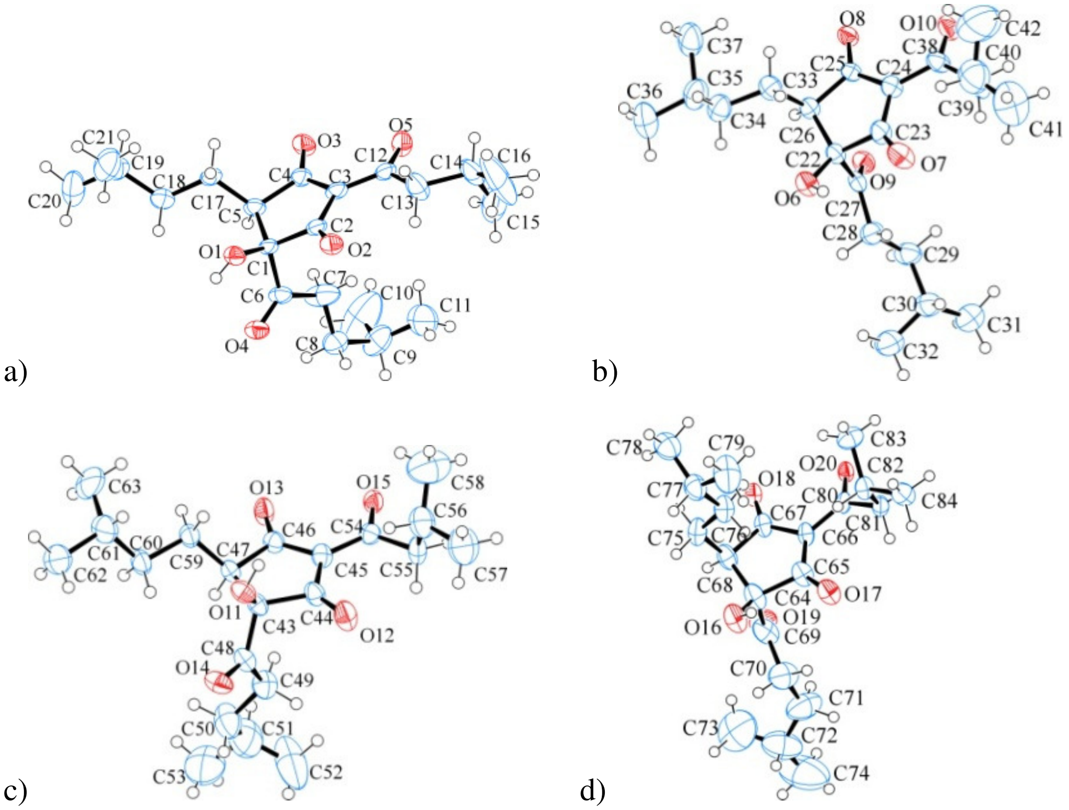

Figure SI-4. The four different anions of compound **1** found in this structure, exhibiting different conformation of the same chirality.

<u>SI3.2 X-ray structure of Compound -1a, potassium salt of (–)-cis n- tetrahydro iso α-acid</u>

Details of the structure for compound (**2**) are similar to that of (**1**), except that it possesses the opposite handedness.

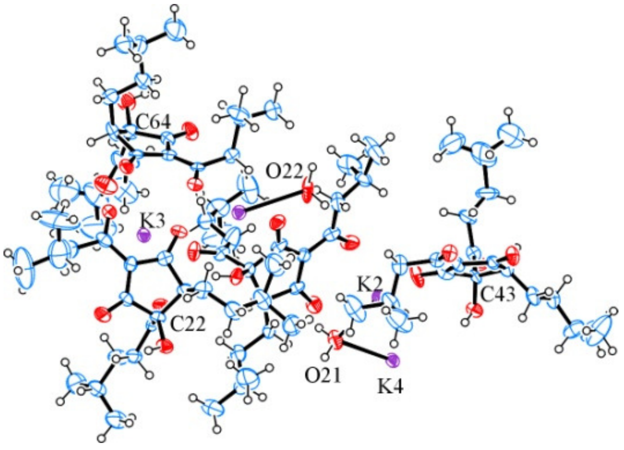

Figure SI-5. ORTEP representations of the asymmetric unit in the structure for compound **-1a** with displacement ellipsoids at the 50% probability level.

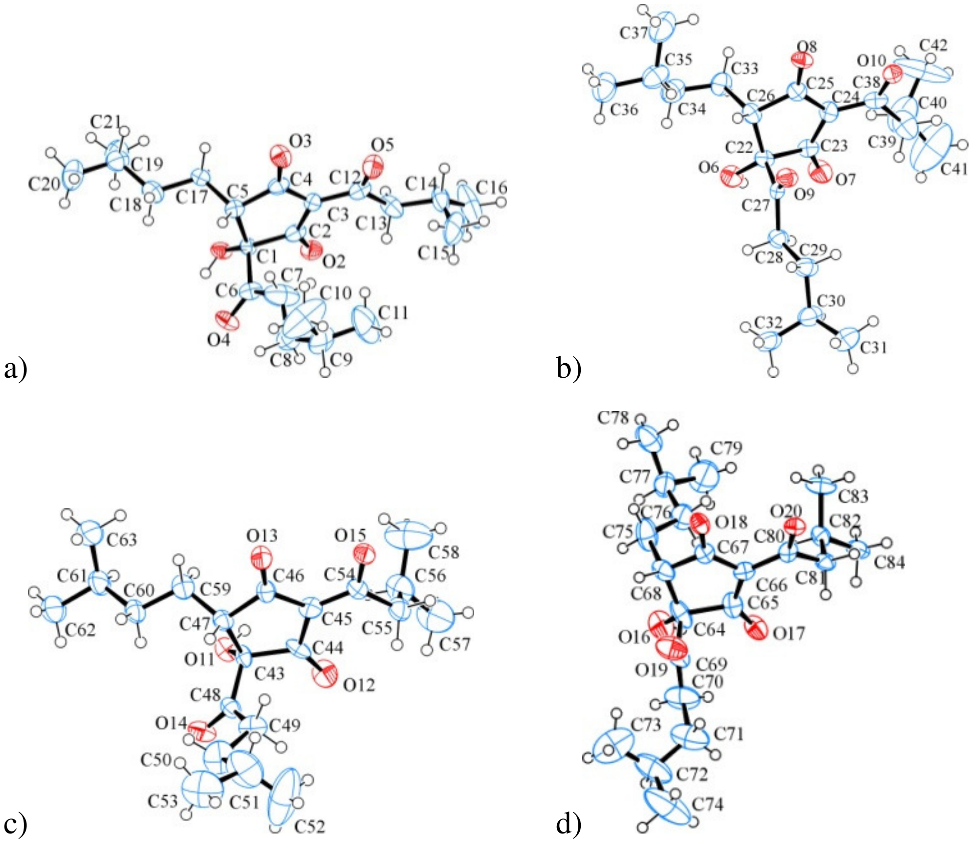

Figure SI-6. The four different anions of compound (**-1**) found in this structure, exhibiting different conformation of the same chirality.

<u>SI3.3 X-ray structure of Compound 2b, (+)-cis co-tetrahydro iso α-acid * cinchonidine</u>

One disordered ^{t}BuOMe molecule was found in addition to the four molecules shown below, the disorder of which causes further disorder in the structure (not demonstrated in Figures SI-7 or SI-8). Table SI-7 summarizes the hydrogen bonds.

Table SI-7. Hydrogen bonds for compound **2b** [Å and °].

D-H...A	d(D-H)	d(H...A)	d(D...A)	<(DHA)
O(1)-H(1A)...N(2)#1	0.84	1.95	2.762(3)	161.2
O(6)-H(6)...O(2)#2	0.84	1.87	2.687(2)	164.7
N(1)-H(1B)...O(3)	0.93	2.03	2.755(3)	133.3
N(1)-H(1B)...O(5)	0.93	2.06	2.824(3)	138.6
O(7)-H(7E)...O(10)	0.84	2.05	2.566(3)	119.1
O(12)-H(12)...N(4)#3	0.84	1.87	2.703(3)	175.0
N(3)-H(3)...O(8)	0.93	2.02	2.746(3)	133.8
N(3)-H(3)...O(11)	0.93	2.09	2.790(3)	130.7

Symmetry transformations used to generate equivalent atoms: #1 x,y-1,z #2 -x,y+1/2,-z #3 -x+2,y+1/2,-z+1

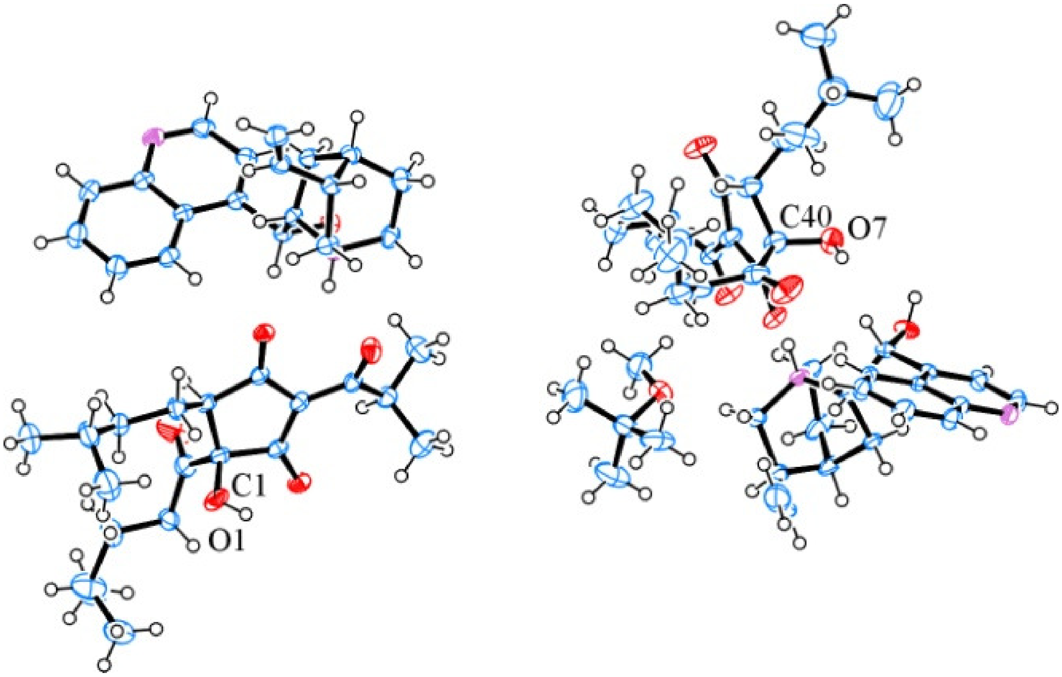

Figure SI-7. ORTEP representations of the asymmetric unit in the structure for compound **2b** with displacement ellipsoids at the 50% probability level.

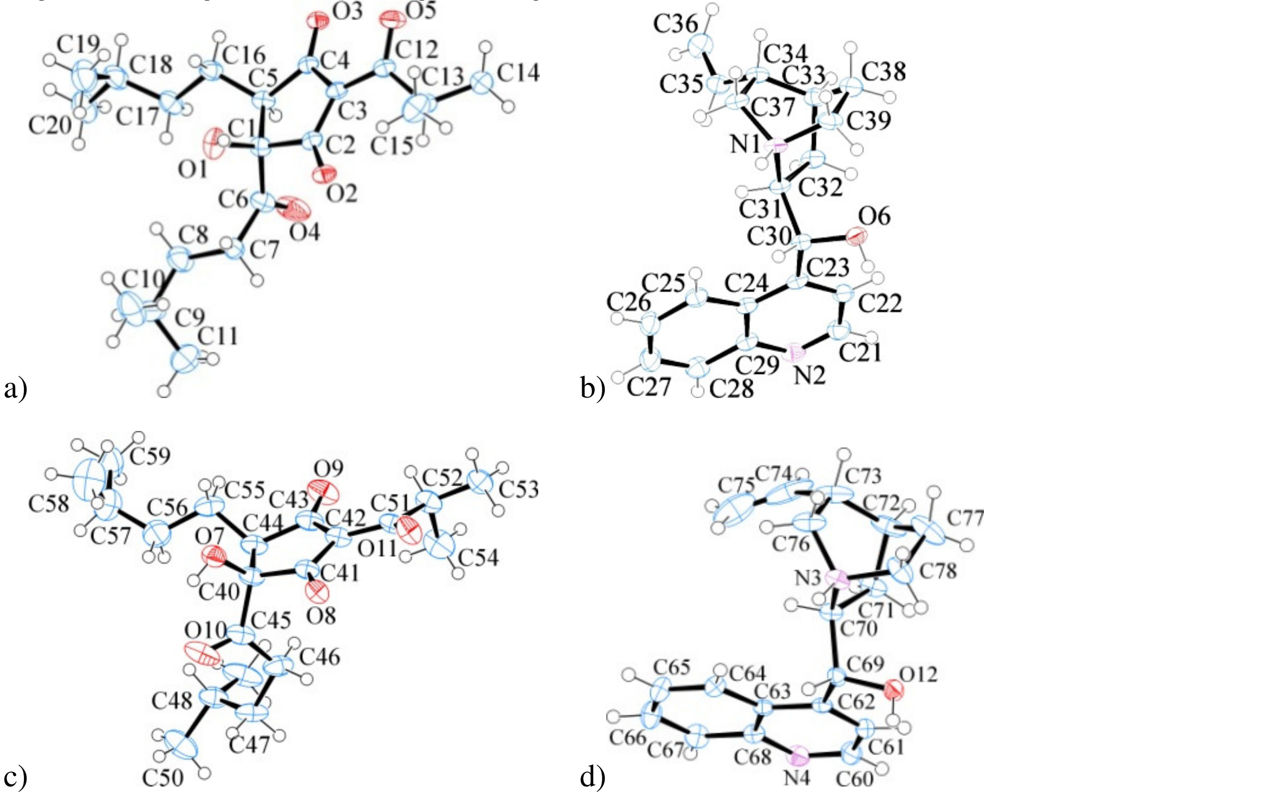

Figure SI-8. a), c): the two different anions of compound **3** found in this structure have slightly different conformations, but exhibit the same chirality; b), d) (–) cinchonidine.

SI3.4 X-ray structure of Compound 3b, (+)-cis ad-tetrahydro iso α-acid * cinchonidine

The structure in SI-9 with details in SI-10 contains disordered ether and isopropyl alcohol at a ratio 28:72 per two salts. The absolute configuration was obtained from comparing the (–) cinchonidine cation to the known absolute configuration.

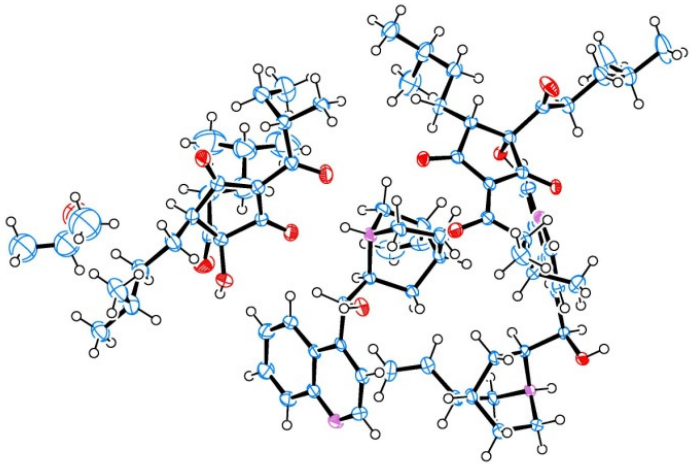

Figure SI-9. ORTEP representations of the asymmetric unit in the structure for compound **3b** with displacement ellipsoids at the 50% probability level. Ether and disorder of isopropyl alcohol are omitted for clarity.

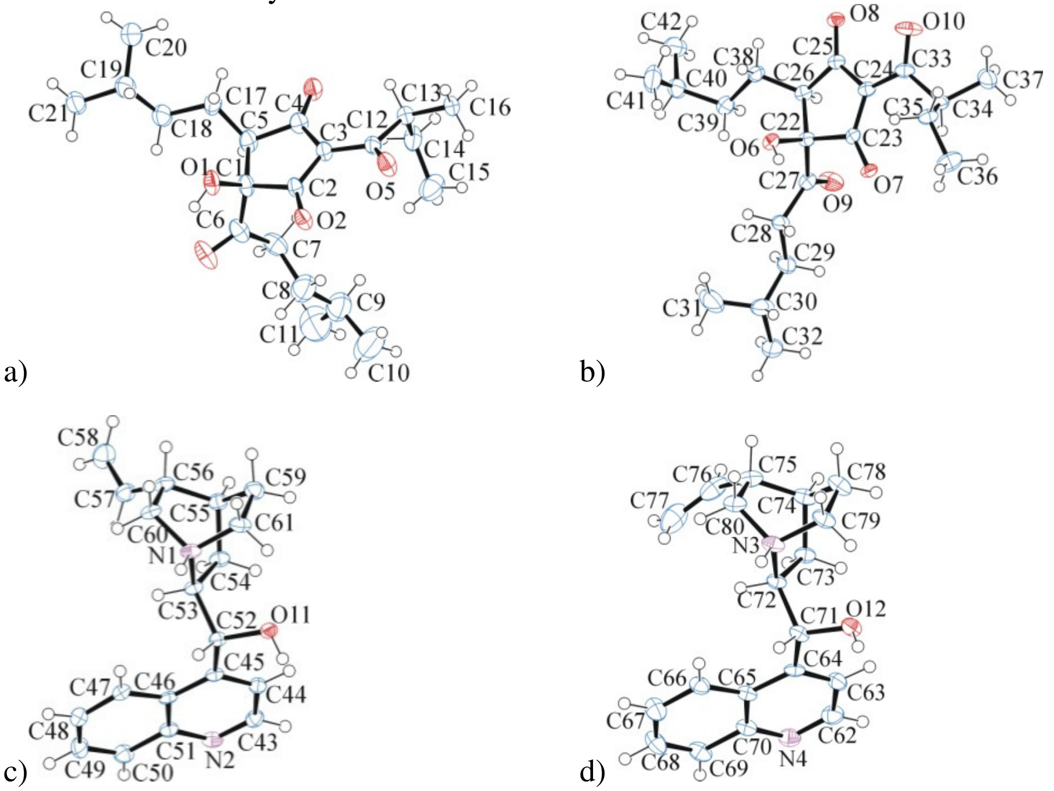

Figure SI-10. a), b) the two different anions of compound **3** found in this figure exhibit slightly different conformations, but possess the same chirality; c), d) (–) cinchonidine.

SI3.5 X-ray structure of Compound 4b, (+)-cis n- iso α-acid * cinchonidine

Several hydrogen bonds are observed which hold this salt structure together (Table SI-8). The absolute configuration was obtained from comparison with the known (–)-cinchonidine for assignment.

Table SI-8. Hydrogen bonds for compound **4b** [Å and °].

D-H...A	d(D-H)	d(H...A)	d(D...A)	<(DHA)
N(1)-H(1)...O(5)	0.93	1.93	2.752(8)	146.4
N(1)-H(1)...O(3)	0.93	2.28	2.942(7)	128.0
O(1)-H(1A)...O(5)#1	0.84	1.92	2.720(6)	159.0
O(6)-H(6)...O(2)#2	0.84	1.91	2.713(7)	158.6

Symmetry transformations used to generate equivalent atoms:
#1 -x,y-1/2,-z+1 #2 -x,y+1/2,-z+1

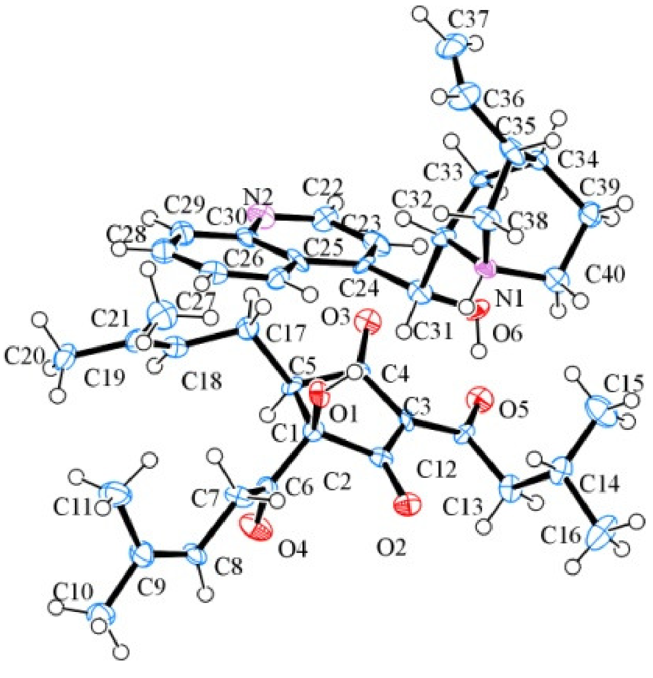

Figure SI-11. ORTEP representations of the asymmetric unit in the structure for compound **4b** with displacement ellipsoids at the 50% probability level.

SI3.6 X-ray structure of Compound 5c, (–)-humulone * (1R, 2R)-1,2-diaminocyclohexane

Upon cooling, the thin and very fragile needle shaped crystals of **5c** disintegrated, where cause and exact temperature for the problem are not known. We thus choose to collect the data at room temperature (19.4^{o}C) which then left us with less scattering, more diffuse background and hence, unsatisfying statistical data agreement factors (R_{int}). The salt (Figure SI-12) exhibits several hydrogen bonds (Table SI-9), notably inter-molecularly O1-N1, intra-molecularly O3-O5, N2-O4. Chiral centers: C22: R, C27: R (reference molecule with known chirality), C1: S.

Table SI-9. Hydrogen bonds for **5c** [Å and °].

D-H...A	d(D-H)	d(H...A)	d(D...A)	<(DHA)
N(1)-H(1A)...O(4)#1	0.86	2.48	3.085(9)	127.6
N(1)-H(1B)...O(1)	0.86	2.56	3.413(10)	172.5
N(2)-H(2A)...O(1)#2	0.89	2.10	2.810(7)	136.5
N(2)-H(2A)...O(2)#2	0.89	2.11	2.865(8)	141.5
N(2)-H(2B)...O(4)#3	0.89	2.12	2.974(8)	159.4
N(2)-H(2C)...O(4)	0.89	1.96	2.847(8)	171.6
N(2)-H(2C)...O(1)	0.89	2.65	3.172(9)	118.3
O(1)-H(1C)...N(1)#2	0.82	1.99	2.765(8)	157.4
O(3)-H(3)...O(5)	0.82	1.66	2.414(9)	152.0

Symmetry transformations used to generate equivalent atoms:
#1 x+1,y,z #2 x-1/2,-y+1/2,-z #3 x+1/2,-y+1/2,-z

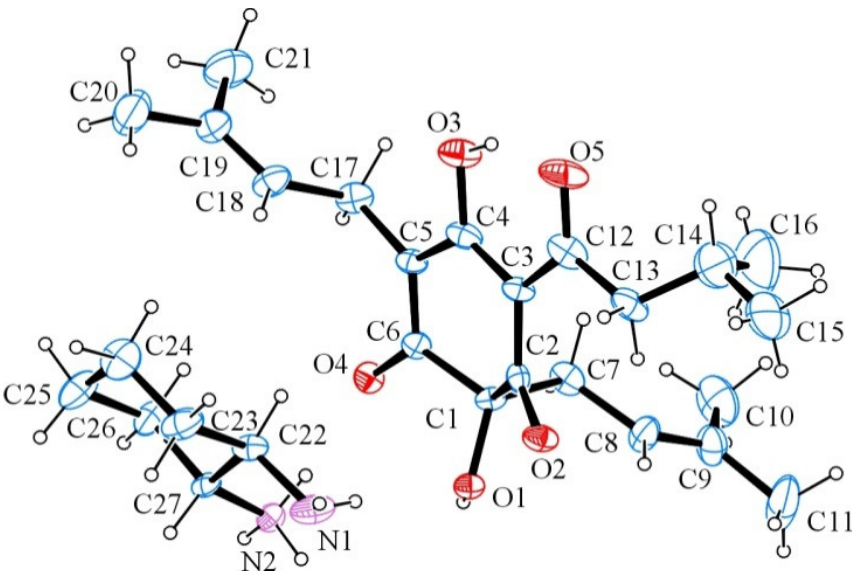

Figure SI-12. ORTEP representations of the asymmetric unit in the structure for compound **5c** with displacement ellipsoids at the 50% probability level.

SI3.7 X-ray structure of Compound 6d, (–)-trans-iso α-acid * cinchonine

The absolute structure was first set to match the known enantiomer of (+) cinchonine. The presence of chloroform (disorder of which not shown in Fig. SI-13) allowed the use of anomalous scattering to determine the absolute structure. The correct assignment was confirmed by the Flack-enantiopole parameter of 0.02(6).

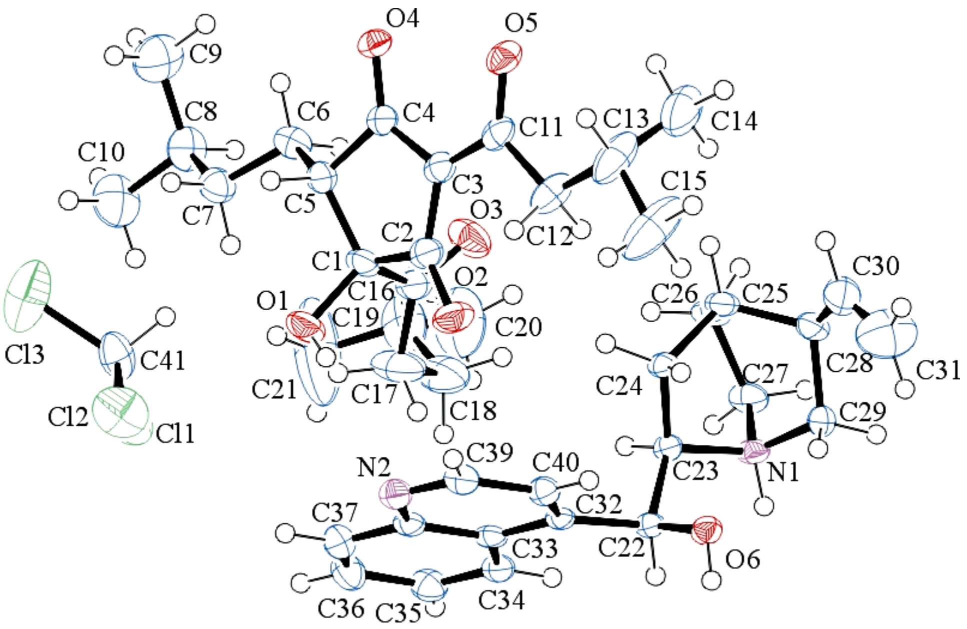

Figure SI-13. ORTEP representations of the asymmetric unit in the structure for compound **6d** with displacement ellipsoids at the 50% probability level. Disorder of solvent omitted for clarity.

References

S1. P. L. Ting, H. Goldstein, *J. Am. Soc. Brew. Chem.* **1996**, 54, 103-109.

S2. B. Carrol, G. Darland, A. Desai, V. Konda, C. J. Dahlberg, J. Urban, *US Patent Application* **2012**, WO2012058649

S3. M. Verzele, D. De Keukeleire, *Chemistry and Analysis of Hop and Beer Bitter Acids*. Elsevier, Amsterdam, **1991**.

S4. Y. Ito, *Journal of chromatography*, **2005**,1065, 145-168.

S5. G. F. Pauli, S. M. Pro, J. B. Friesen, *J. Nat. Prod.* **2008**, 71, 1489-1508.

S6. Y. Yuan, B. Wang, L. Chen, H. Luo, D. Fisher, I. A. Sutherland, Y. Wei, *J. Chromatogr.* A. **2008**, 1194, 192-198.

S7. C. J. Dahlberg, J. S. Traub, M. L. Tripp, J. S. Bland, B. J. Carroll, *J. Sep. Sci.* **2010**, 33, 2828–2832.

S8. A. Khatib, E. G. Wilson, M. Supardi, R. Verpoorte, *Food Chemistry* **2010**, 119, 354-357.

S9. C. J. Dahlberg, G. Harris, J. Urban, M. L. Tripp, J. S. Bland, B. J. Carroll. Isolation of bitter acids from hops (Humulus lupulus L.) using countercurrent chromatography, J. *Sep. Sci*. **2012**, 35, 1182-1189.

S10. A. C. HermansLokkerbol, A. C. Hoek, R. Verpoorte, *Journal of Chromatography* A **1997**, 771, 71-79.

S11. B. D. A. Hook, W. Dohle, P. R. Hirst, M. Pickworth, M. B. Berry, K. I. A Booker-Milburn, J. *Org. Chem*. **2005**, 70, 7558-7564.

S12. *Bruker APEX2 (Version 2.1-4), SAINT (version 7.34A), SADABS (version 2007/4)* BrukerAXS Inc, Madison, Wisconsin, **2007**.

S13. A. Altomare, C. Burla, M. Camalli, L. Cascarano, C. Giacovazzo, A. Guagliardi, A. G. G. Moliterni, G. Polidori, R. J. Spagna, *Appl. Cryst*. **1999**, 32, 115-119.

S14. A. Altomare, G. Cascarano, C. Giacovazzo, A. J. Guagliardi, *Appl. Cryst*. **1993**, 26, 343-350.

S15. G. M. Sheldrick, *SHELXL-97: Program for the Refinement of Crystal Structures.* Univ. of Gottingen, Germany, **1997**.

S16. S. Mackay, C. Edwards, A. Henderson, A. C. Gilmore, N. Stewart, K. Shankland, A. Donald, *MaXus: a computer program for the solution and refinement of crystal structures from diffraction data.* University of Glasgow, Scotland, **1997**.

S17. D. Waasmaier, A. Kirfel, *Acta Crystallogr.* A **1995**, 51, 416-430.
